# Nipple Melanoma: A Case Report and Review of the Literature

**DOI:** 10.1159/cde/abvag006

**Published:** 2026-08-14

**Authors:** Ana Ortiz González, Marina Torrent Gracia, Claudia Alejandra Capozzi, Sebastián Ortiz Reina, José Balsalobre Yago, Antonio Piñero Madrona, Pablo Cerezuela-Fuentes

**Affiliations:** a Department of Pathology, Hospital General Universitario “Santa María del Rosell”, Complejo Hospitalario de Cartagena, Cartagena, Spain; b Department of Dermatology, Hospital General Universitario “Santa María del Rosell”, Complejo Hospitalario de Cartagena, Cartagena, Spain; c Medical Oncology Department, Hospital General Universitario “Santa Lucía”, Complejo Hospitalario de Cartagena, Cartagena, Spain; d Head of Breast Surgery Unit, Surgery Department, Hospital Clínico Universitario “Virgen de la Arrixaca”, InstitutoMurciano de Investigación Biosanitaria (IMIB) Ciudad de Murcia, Professor of Surgery Universidad de Murcia, Murcia, Spain; e Medical Oncolgy Department, Hospital Clínico Universitario “Virgen de la Arrixaca”, Ciudad de Murcia, InstitutoMurciano de Investigación Biosanitaria (IMIB), Murcia, Spain

**Keywords:** Nipple melanoma, Case report, Review

## Abstract

**Introduction:**

Primary melanoma of the nipple-areolar complex (NAC) is an exceptionally rare malignancy and may pose significant diagnostic challenges because of its overlap with other breast and cutaneous neoplasms. We report a case of primary NAC melanoma initially misdiagnosed as an undifferentiated sarcoma.

**Case Presentation:**

A 93-year-old woman presented with an 8-month history of a bleeding, ulcerated, exophytic lesion of the left nipple. Imaging demonstrated a suspicious NAC mass with radiologically enlarged ipsilateral axillary lymph nodes, while fine-needle aspiration was negative. Core biopsy suggested an undifferentiated sarcoma. Following modified radical mastectomy and axillary lymph node dissection, histopathological and immunohistochemical examination established the diagnosis of ulcerated invasive superficial spreading melanoma of the NAC (Breslow ≥60 mm, pT4bN0M0, and AJCC stage IIC). All 15 lymph nodes were negative. No adjuvant treatment was administered, and the patient remains disease-free 26 months after diagnosis.

**Conclusion:**

NAC melanoma should be considered in the differential diagnosis of persistent nipple lesions. Extensive histopathological assessment and comprehensive immunohistochemical studies are essential, particularly in poorly differentiated tumors with heterogeneous marker expression. Early recognition and multidisciplinary management may help avoid diagnostic delays and inappropriate treatment.

## Introduction

Melanoma is a malignant neoplasm that typically affects sun-exposed skin, although it may arise in less frequent locations such as mucosal surfaces, the uvea or acral sites [1]. Although the incidence of cutaneous melanoma has increased in recent years [2], primary melanoma of the nipple-areolar complex (NAC) is exceptionally rare, with only small case series and isolated reports published since the late 1970s [3]. Its diagnosis may be delayed because of its clinical similarity to pigmented Paget disease, breast carcinoma, and various benign lesions (nevi, seborrheic keratoses, and melanosis of the areola), among others [4]. Histopathological confirmation is essential and may be challenging in the presence of atypical immunohistochemical profiles. We present a clinical case of primary nipple melanoma in a 93-year-old woman, initially diagnosed as an undifferentiated sarcoma.

## Case Presentation

A 93-year-old woman, who had been independent in basic and instrumental activities of daily living until symptom onset, with no known drug allergies and no relevant past medical history, and with a family history of a sister who died at 76 years of age from breast cancer, presented with a tumor of the left nipple of approximately 8 months of duration. The lesion was exophytic, nodular, pedunculated, violaceous, firm to hard, ulcerated and bleeding, measuring 3–4 cm in diameter and attached to the nipple by a 1-cm pedicle. No palpable axillary lymphadenopathy was detected (Fig. 1).

**Fig. 1.**
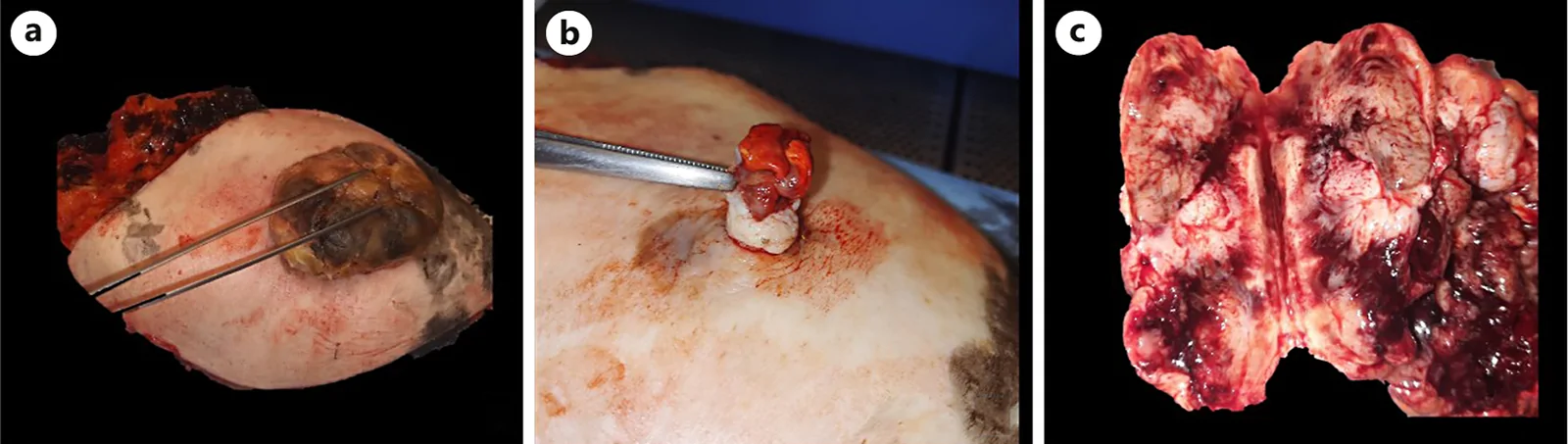
**a** Surgical specimen showing an exophytic tumor mass. **b** Pedicle of the nodular lesion. **c** Macroscopic cross-section of the lesion.

Digital breast tomosynthesis and breast and axillary ultrasound were performed, demonstrating a 44 × 35 mm mass in the left nipple (BI-RADS 5) with at least five ipsilateral lymph nodes at Berg levels I and II, one dominant node measuring 7.4 mm. Fine-needle aspiration of the dominant node was negative for malignant cells. Thoracoabdominal-pelvic CT showed no evidence of distant disease.

Core-needle biopsy of the lesion revealed a monotonous neoplasm composed of cells with scant cytoplasm and pleomorphic nuclei with vesicular chromatin and small nucleoli, arranged in dense aggregates with minimal stroma. Immunophenotyping showed positivity for vimentin and other mesenchymal markers (SATB2, WT1, desmin, and TLE1) and negativity for epithelial markers (cytokeratins), neuroendocrine, lymphoid, neuroectodermal (S100) and mammary markers. With a diagnosis suggestive of undifferentiated sarcoma, a left modified radical mastectomy with axillary lymph node dissection was performed.

Histopathological examination of the surgical specimen revealed, in the epidermis and at the dermoepidermal junction, a proliferation of atypical melanocytes with nuclear pleomorphism, numerous mitotic figures and pagetoid spread, extending into the dermis. In the dermis, medium-sized cells with scant eosinophilic cytoplasm, a diffuse growth pattern, occasional intracytoplasmic melanin pigment, and large pleomorphic, hyperchromatic nuclei with small nucleoli were observed. Fascicular areas composed of spindle cells, foci with markedly enlarged (“monster”) nuclei, and extensive areas of hemorrhage and necrosis were also identified.

Given the suspicion of melanoma, immunohistochemical studies (Melan A, S100, HMB45, PRAME, and tyrosinase) were performed and showed positivity at the dermoepidermal junction and patchy positivity within the dermis (Fig. 2). Histological and immunohistochemical evaluation of the ipsilateral lymph node chains was negative.

**Fig. 2.**
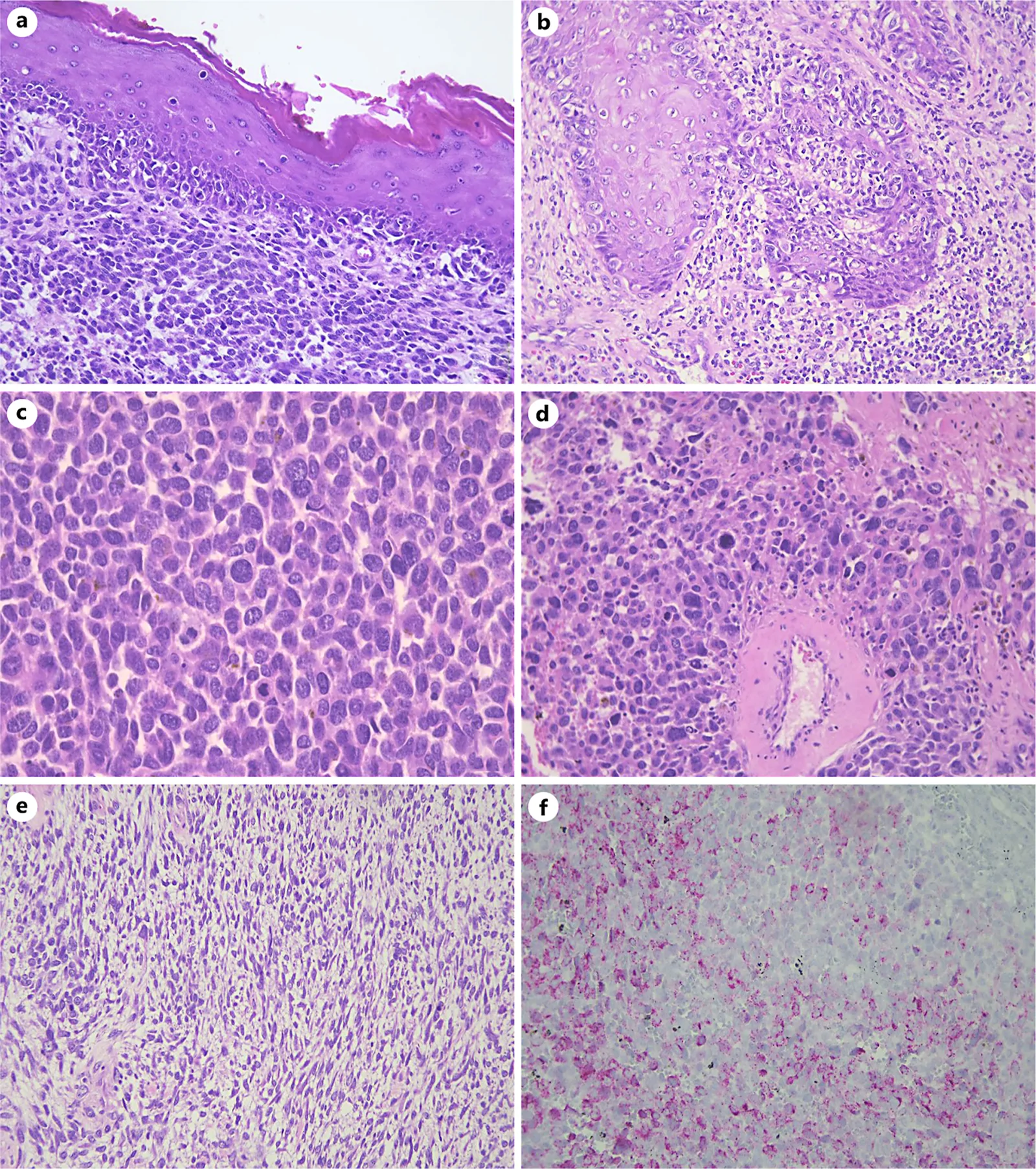
**a**, **b** Intraepidermal component with pagetoid growth. **c** Intradermal component of the tumor. **d** Areas with markedly enlarged (“monster”) nuclei. **e** Areas with fascicular growth. **f** Patchy HMB45 expression.

The final histopathological diagnosis was invasive superficial spreading melanoma of the NAC, measuring 55 mm in maximum macroscopic diameter, ulcerated, with a Breslow thickness of at least 60 mm, Clark level IV, and a mitotic rate of 7 mitoses/mm^2^, without vascular invasion, microsatellitosis or neurotropism, and with tumor-free surgical margins. Areas of partial regression were identified. All 15 resected axillary lymph nodes were negative, and the final stage according to the 8th edition of the AJCC was IIC (pT4b pN0 M0).

After review by a Multidisciplinary Tumor Board, the medical and radiation oncology teams decided against adjuvant treatment. At follow-up in May 2026, 26 months after diagnosis, the patient remained disease-free.

For the reporting of this clinical case, the CARE Checklist, provided as online supplementary material (for all online suppl. material, see https://doi.org/10.1159/cde/abvag006), has been considered.

## Discussion

The incidence of melanoma has increased in recent years; the projected crude incidence rate of cutaneous melanoma in Spain for all sexes in 2025 is 19.0 cases per 100,000 inhabitants (95% CI, 15.2–23.8) [5]. Melanoma is the cutaneous neoplasm with the second highest lethality; however, earlier detection, the implementation of standardized surgical techniques, and the development of new treatments such as immunotherapy and targeted therapy have led to improved survival outcomes [6].

As the patient was initially assessed by the breast surgery team, and given both her family history of breast cancer and the rarity of primary melanoma involving the nipple, the lesion was approached without the involvement of the Dermatology Department, and dermoscopic examination was not performed. Furthermore, as melanoma was not initially considered in the differential diagnosis, the immunohistochemical panel was not expanded to include specific melanocytic differentiation markers.

Primary melanomas of the skin of the breast account for 0.28%–3.8% of all invasive cutaneous melanomas [7–9]. Primary melanoma of the NAC is considered an extremely rare variant, representing approximately 12% of all cutaneous melanomas of the breast [3].

The low frequency of invasive and in situ melanomas involving the NAC remains poorly understood. It may be related, at least in part, to the relatively low ultraviolet exposure of this anatomical site and to its particular clinical and anatomical characteristics. The true incidence of melanomas arising from the cutaneous surface of the breast is likely underrepresented, as many series classify the skin of the trunk as a single location [10, 11]; at most, some distinguish between the anterior and posterior chest [12]. In addition, the reported percentages often derive from very small and heterogeneous series that include both primary and metastatic lesions [13].

Furthermore, while some authors have proposed that primary melanoma of the breast (PMB) represents a metastatic event, others have argued that it constitutes a distinct primary lesion of the breast [14]. Nevertheless, the pathogenesis of these exceptionally rare tumors remains poorly understood, and their classification continues to be debated. Cases reported under the designation of primary melanoma of the breast parenchyma are exceedingly rare, and the underlying mechanism responsible for this presentation remains unclear. Consequently, the nosological status of PMB remains uncertain, and whether it represents a distinct clinicopathological entity continues to be debated [15].

Haagensen [16] was the first to report a melanoma of the NAC in the second edition of *Diseases of the Breast* (1971), whereas the first series of NAC melanomas was subsequently reported by Papachristou [3]. The clinical presentation of NAC melanoma is heterogeneous (see Table 1, “Characteristics of NAC melanomas”; this table excludes, except in the case of series, melanomas arising in non-NAC breast skin or lesions that, after review and discussion by the authors, were considered unlikely to represent true NAC melanoma) and should be considered in the differential diagnosis of pigmented lesions of this structure in individuals of any sex. It is important to recognize that benign pigmented lesions such as nevi, seborrheic keratoses, and areolar melanosis are more common, and that other malignant cutaneous neoplasms, including cutaneous metastases, Paget disease of the breast and its uncommon pigmented variant – characterized by pagetoid spread of malignant epithelial cells associated with epidermal pigmentation – must also be taken into account, which further complicates the differential diagnosis with primary breast melanoma [17–19].

**Table 1.** Characteristics of melanomas of the NAC

Reference/Report type	*N*	Sex	Age	Breslow (mm)	Mitoses/mm^2^	Ulceration/Clark	Types of melanoma	BRAF	Stage	Breast surgery	Axillary surgery	Follow up/status
Papachristou et al. [3] (1979)	14 (4 in the nipple)	2 men	Median age 32 (28–62)^a^			NR/1 case IV; the rest NR			4/14 axillar (+), stage III (all died within <3 years)	9 RM	ALND	>5 years
Case series		2 women							10/14 axillar (−), stage II (alive at 5 years)	4 MRM		
										1 ERM		
Roses et al. [9] (1979)	21: 3 melanomas of the NAC	3 men	Mean age 45 (24–72)^a^			NR/Clark: Level II: 6 Level III: 9 Level IV: 4 Level V: 1 NR: 1	1 NR		I and II	18 WE	12 ALND	Mean: 57 months (1–144)^a^
Case series							18 SSM			2 RM	Rest: NR	
							2 NM			1 NR		
D’Aiuto et al. [20] (1991)	1	Woman	53	NR	NR	IV	NR	NR	NR	WE	ALND	Alive at 5 years
Single case report												
Lin et al. [21] (2007)	1	Woman	86	NR	NR	NR/NR	NM	NR	NR	WE	NR	Alive at 6 months
Single case report												
Kinoshita et al. [22] (2007)	1	Woman	42	4	NR	Yes/NR	NR	NR	III	WE	ALND	Alive at 15 months
Single case report												
Mourmouras et al. [23] (2011)	1	Woman	47	5.5	20	Yes/IV	AM	NR	IV	WE	ALND	Died at 16 months
Single case report												
Duman et al. [21] (2015)	1	Woman	45	11	1	Yes/IV	NR	Native	T4bN3M0	MRM	SLNB and ALND	Died at 4 years
Single case report												
D’Antonio et al. [24] (2017)	1	Woman	58	1.5	NR	Yes/NR	NR	NR	T2bN2a	WE	ALND	Alive at 6 months
Single case report												
Nagata et al. [25] (2018)	1	Woman	59	12	NR	Yes/IV	NM	NR	T4bN1bM0	M	SLNB and ALND	Alive at 7 years
Single case report												
Moukit et al. [26] (2021)	1	Woman	54	SSM in situ	NR	NA/I	SSM		0	WE	ALND	Alive at 12 months
Single case report												
Dehner et al. [18] (2024)	10	5 women	Median age: 55.5 (29–66)^a^	In 6 cases: 0.2–3.5 mm	Median <1, except in case 7 (8 mm)^a^	None/NR^a^	NR	NR		WE. Two patients ultimately underwent mastectomy.^a^	6 with SLNB^a^	Median follow up: 19 months (1–183 months)^a^
		5 men^a^		In 4: melanoma in situ^a^								
		Man	56	Melanoma in situ	NA	NA/NA	NA		0	WE	NA	Alive at 58 months
		Man	57	1.3	0	No/NR			pT2a	WE	SLNB	Alive at 13 months
		Man	66	0.2	0	No/NR			pT1a	WE	NR	Alive at 13 months
		Woman	29	0.9	0	No/NR			pT1b	WE	SLNB	Alive at 55.5 months
		Man	63	2.0	1	No/NR			pT2a	WE	SLNB	Alive at 4 months
Case series		Woman	55	Melanoma in situ	NA	NA/I	NA		0	WE	NR	Alive at 97 months
		Woman	42	3.5	8	No/NR			pT3aN1	M	NR	Died at 25.5 months
		Man	47	3.1	3	No/NR			pT3a	WE	SLNB	Alive at 183 months
		Woman	56	Melanoma in situ	NA	NA/I	NA		0	WE	NA	Alive at 1 months
		Woman	42	Melanoma in situ	NA	NA/I	NA		0	M	SLNB	Alive at 3 months
Giudice et al. [27] (2024)	1	Woman	43	Melanoma in situ	NA	NA/I	NA	Native	0	M	SLNB^b^	NR
Single case report												
Hines et al. [28] (2024)	11: 1 melanoma of the NAC	91% women^a^	40 (20–73)^a^	Mediana 0.55 (0.34-1-1)^a^	NR	NC/NR	NR	NR	NR	All underwent WE	2 with SLNB^a^	Median 8.8 years^a^
MES												
Case series												
The present case	1	Woman	93	60	7	Yes/IV	SSM	NR	pT4bN0M0	MRM	ALND	Alive at 19 months
Single case report												

ALND, axillary lymph node dissection; ERM, extended radical mastectomy; M, mastectomy; MRM, modified radical mastectomy; RM, radical mastectomy; NA, not applicable; NAC, nipple-areolar complex; NR, not reported; SLNB, sentinel lymph node biopsy; WE, wide excision; AM, acral melanoma; NM, nodular melanoma; SSM, superficial spreading melanoma.

^a^ Aggregate data from the series.

b Associated ductal carcinoma *in situ* in the breast.

With respect to sex distribution, an early report suggested a higher frequency in men [3]. However, when considering other publications, including more recent reports of NAC melanoma, the condition appears to be more frequent in women [20–27, 29]. The cohort reported by Dehner et al. [18] showed a roughly equal distribution between sexes, underscoring the importance of considering primary melanoma in the differential diagnosis of pigmented nipple-areolar lesions in both men and women.

Regarding the depth of cutaneous invasion, Roses et al. [9] noted that most tumors corresponded to Clark levels II or III; however, that series included 21 melanomas involving the skin of the breast, of which only 3 arose in the NAC (see Table 1). In the present case, the Clark level was consistent with a thick lesion.

The age of the patient described is unusual; in most published series and case reports, individuals are considerably younger [3, 9, 18, 20–27, 30, 31], and to our knowledge this is the first reported case of NAC melanoma in a person older than 90 years. In the present case, the clinical and radiological assessment did not progress beyond the identification of a malignant neoplasm of the NAC. The histopathological diagnosis of PMB is complex and relies on a combination of morphologic and immunohistochemical features. The marked cytologic and architectural variability with which these tumors may present makes immunohistochemistry essential to distinguish melanomas from metastases of extramammary neoplasms and from epithelial, hematologic, mesenchymal and neural tumors. S100 is the most sensitive marker for melanoma (with a sensitivity greater than 90%). Melan A and HMB45 are also useful immunohistochemical markers in the diagnosis of melanoma [30, 31].

However, in highly undifferentiated lesions such as the one presented, melanocytic markers may be negative (S100-negative), and in the setting of vimentin and desmin positivity, it may initially appear reasonable not to perform additional melanocytic markers such as HMB45 or PRAME. With this immunohistochemical profile, confusion between melanoma and sarcoma is common and represents a relatively frequent diagnostic challenge [32]. Comprehensive examination of the entire lesion is therefore crucial to establish a definitive diagnosis. The lesion might have been initially diagnosed as melanoma if a wider excisional biopsy had been performed, thus preventing sampling error by encompassing areas that preserved antigenic expression for immunohistochemical markers of melanocytic differentiation, which would likely have modified the subsequent therapeutic approach. In this case, the negative S100 result in the presurgical biopsy is most likely attributable to tumor heterogeneity and the use of a core needle biopsy. Immunohistochemical analysis of the surgical specimen demonstrated heterogeneous S100 protein expression, with alternating positive and negative areas. A similar pattern was observed for other melanocytic differentiation markers, including Melan-A, HMB45, PRAME, and tyrosinase. Therefore, the initial negative S100 staining in the incisional biopsy can be explained by sampling of a tumor area lacking S100 expression, reflecting intratumoral immunophenotypic heterogeneity rather than a true absence of melanocytic differentiation.

Regarding treatment, there is some controversy in surgical management between local excision and mastectomy. In the present case, surgery was performed according to the initial histologic diagnosis of sarcoma, with axillary lymph node dissection undertaken because of radiologically detected lymphadenopathy. The earliest series [3] tended to favor mastectomy, whereas more recent series and case reports [9, 18, 20, 22–24, 26, 28, 29] recommend avoiding mastectomy and managing the lesion as an invasive cutaneous melanoma. At this point, it is worth considering that a more aggressive approach in the initial biopsy (perhaps an excisional biopsy) could have provided more complete information about the diagnosis and could have modified the therapeutic treatment.

In any event, the rarity of cutaneous melanoma of the nipple means that no specific guidelines exist to define a standardized surgical approach. Most authors recommend managing both the primary lesion and the axilla according to current melanoma guidelines [28]. Mastectomy does not offer an advantage over wide local excision of the primary lesion. Standard melanoma treatment consists of surgical excision with a safety margin, which is more relevant in prognostic than in cosmetic terms. No statistically significant differences have been demonstrated in 5-year overall or disease-free survival between wider excision (3–5 cm margins) and more conservative excision (1–2 cm margins) [33, 34]. It is currently considered that the optimal surgical margin around a primary invasive cutaneous melanoma with a tumor thickness greater than 2 mm is considered to be 2 cm [34].

With respect to axillary management, sentinel lymph node biopsy is the standard procedure to assess regional nodal involvement in both breast cancer and malignant melanoma, and it contributes to a reduction in treatment-related adverse effects [35]. In our specific case, surgery was planned on the basis of a prior diagnosis of undifferentiated sarcoma with negative cytology but radiologically suspicious axillary involvement; therefore, management of the lesion by modified radical mastectomy with ipsilateral axillary lymph node dissection appears appropriate.

With respect to systemic adjuvant therapy directed at melanoma micrometastases, this approach is indicated in postoperative individuals at high risk of progression to advanced disease [36–38]. In the case presented, after careful multidisciplinary assessment, adjuvant therapy was not recommended, primarily in view of the person’s age, in line with previously reported trends [39].

Regarding prognosis, Papachristou et al. [3] described a generally favorable clinical course for cutaneous NAC melanomas, with axillary involvement conferring an ominous prognosis. At present, tumor thickness is considered the main prognostic factor in primary cutaneous melanoma. In the case reported here, the tumor thickness reached at least 60 mm, which, to the best of our knowledge, represents the first report of NAC melanoma with such an extraordinarily high thickness. Although the clinical characteristics of melanomas at this site remain incompletely defined, in the present case the anatomical location – rather than tumor thickness or histologic subtype – may have a greater influence on prognosis [3]. The patient has had a favorable course, remaining free of recurrence during 26 months of follow-up after surgery.

## Conclusion

The clinical characteristics of melanomas arising in the nipple and areola remain poorly defined because of the scarcity of studies specifically addressing their particular features. Indeed, available incidence data derive from series and case reports that often pool lesions of the breast skin, the NAC and even metastatic lesions, and the very existence of primary breast melanoma has been questioned, underscoring the need for a clearer understanding of its biological basis and clinicopathological classification.

Although melanoma of the NAC is an uncommon entity, it should be considered in the differential diagnosis of pigmented breast lesions in individuals of any sex. Diagnosis relies on histology and immunohistochemistry, and, whenever feasible, the most appropriate diagnostic procedures – particularly excisional biopsy – should be performed to ensure accurate assessment, as limited sampling techniques may lead to misdiagnosis or delay. The optimal surgical approach to both the primary lesion and the axilla appears to align with current standards for cutaneous melanoma, although decisions should be made within Multidisciplinary Tumor Boards and tailored to each patient’s clinical context, including postoperative treatment. Prognosis appears to be influenced, in addition to the usual factors described for primary cutaneous melanoma, by the presence or absence of nodal involvement. We consider it important to share experiences such as the present case in the scientific literature, with the aim of expanding the evidence available on NAC melanoma and helping to prevent future delays in diagnosis when clinicians assess individuals with persistent cutaneous changes in the NAC.

## Patient Perspective

The patient was previously informed of all procedures to be performed, as well as of any uncertainties arising regarding the diagnosis. Likewise, she actively participated in the therapeutic and follow-up decision-making processes.

## Statement of Ethics

This case report did not require formal approval by an institutional review board or ethics committee, as per local and institutional guidelines for single-patient case descriptions that do not contain experimental interventions. Written informed consent was obtained from the patient for publication of this case report, including all clinical details and any accompanying images. All identifying information has been removed to protect the patient’s privacy. No personal data that could reveal the patient’s identity are included in this publication. This report was prepared in accordance with the ethical principles outlined in the Declaration of Helsinki and with the publication policies of Karger’s *Case Reports in Dermatology* regarding patient consent and data protection.

## Conflict of Interest Statement

The authors has/have no conflicts of interest to declare.

## Funding Sources

The authors declare no funding was obtained or used for this study.

## Author Contributions

Ana Ortiz González: conceptualization, investigation, writing – original draft, review, and editing. Marina Torrent Gracía: investigation, resources, writing – review and editing. Claudia Alejandra Capozzi: investigation, visualization, writing – review and editing. Sebastián Ortiz Reina: investigation, writing – review and editing. José Balsalobre Yago: investigation, validation, writing – review and editing. Antonio Piñero Madrona: methodology, writing – review and editing. Pablo Cerezuela-Fuentes: conceptualization, methodology, investigation, resources, validation, visualization, writing – original draft, review, and editing, supervision, and project administration.

## Data Availability

All data supporting this case report are included in the article. No new datasets were generated; the comparative table is based solely on previously published data. Further anonymized clinical details may be available from the corresponding author upon reasonable request, provided that patient privacy and confidentiality are fully protected.

## References

[1] Centeno PP , Pavet V, Marais R. The journey from melanocytes to melanoma. Nat Rev Cancer. 2023;23(6):372–90. 10.1038/s41568-023-00565-7 37095242

[2] Arnold M , Singh D, Laversanne M, Vignat J, Vaccarella S, Meheus F, . Global burden of cutaneous melanoma in 2020 and projections to 2040. JAMA Dermatol. 2022;158(5):495–503. 10.1001/jamadermatol.2022.0160 PMC8968696 35353115

[3] Papachristou DN , Kinne D, Ashikari R, Fortner JG. Melanoma of the nipple and areola. Br J Surg. 1979;66(4):287–8. 10.1002/bjs.1800660424 455001

[4] Cinotti E , Galluccio D, Tognetti L, Habougit C, Manganoni AM, Venturini M, . Nipple and areola lesions: review of dermoscopy and reflectance confocal microscopy features. J Eur Acad Dermatol Venereol. 2019;33(10):1837–46. 10.1111/jdv.15727 31166040

[5] Red Española de Registros de Cáncer. Estimaciones de la incidencia de cáncer en España, 2025 [Internet]. REDECAN; 2025 [cited 2025 Aug 31]. Available from: https://redecan.org.

[6] Garbe C , Amaral T, Peris K, Hauschild A, Arenberger P, Basset-Seguin N, . European consensus-based interdisciplinary guideline for melanoma. Part 1: Diagnostics - Update 2024. Eur J Cancer. 2025;215:115152. 10.1016/j.ejca.2024.115152 39700658

[7] Ariel IM , Caron AS. Diagnosis and treatment of malignant melanoma arising from the skin of the female breast. Am J Surg. 1972;124(3):384–90. 10.1016/0002-9610(72)90047-5 4115719

[8] Greenberg BM , Hamilton R, Rothkopf DM, Balk M, Clark WH Jr, LaRossa D. Management of cutaneous melanomas of the female breast. Plast Reconstr Surg. 1987;80(3):409–15. 10.1097/00006534-198709000-00011 3628570

[9] Roses DF , Harris MN, Stern JS, Gumport SL. Cutaneous melanoma of the breast. Ann Surg. 1979;189(1):112–5. 10.1097/00000658-197901000-00021 PMC1396944 758856

[10] Howard MD , Wee E, Wolfe R, McLean CA, Kelly JW, Pan Y. Anatomic location of primary melanoma: survival differences and sun exposure. J Am Acad Dermatol. 2019;81(2):500–9. 10.1016/j.jaad.2019.04.034 31009667

[11] Shannon CM , Mehta NK, Li H, Nguyen SA, Koochakzadeh S, Elston DM, . Anatomic region of cutaneous melanoma impacts survival and clinical outcomes: a population-based analysis. Cancers (Basel). 2023;15(4):1229. 10.3390/cancers15041229 PMC9954057 36831571

[12] Tas F , Erturk K. Trunk melanomas: no survival differences between lesion sites. Postgrad Med J. 2022;134(6):609–15. 10.1080/00325481.2022.2080380 35584552

[13] Kurul S , Taş F, Büyükbabani N, Mudun A, Baykal C, Camlica H. Different manifestations of malignant melanoma in the breast: a report of 12 cases and a review of the literature. Jpn J Clin Oncol. 2005;35(4):202–6. 10.1093/jjco/hyi068 15845569

[14] Rassouli M , Voutsadakis IA. Primary noncutaneous malignant melanoma of the breast. Breast J. 2016;22(6):688–91. 10.1111/tbj.12661 27488963

[15] Narvaez-Rojas AR , Linhares S, Sedighim S, Klingbeil KD, Milikowski C, Elgart G, . Is primary breast melanoma a true pathological entity? The argument against it. Heliyon. 2024;10(18):e37224. 10.1016/j.heliyon.2024.e37224 PMC11414497 39309840

[16] Haagensen CD . Diseases of the breast. 3rd ed. Philadelphia: WB Saunders; 1986; p. 352–6.

[17] Koh J , Lee J, Jung SY, Kang HS, Yun T, Kwon Y. Primary malignant melanoma of the breast: a report of two cases. J Pathol Transl Med. 2019;53(2):119–24. 10.4132/jptm.2018.10.18 PMC6435985 30472815

[18] Dehner CA , Johnson EF, Peters MS, Guo RR. Primary melanoma of the nipple: report of 10 cases including coexistence with Paget’s disease. Pathol Res Pract. 2024;253:155058. 10.1016/j.prp.2023.155058 38181578

[19] Brugués A , Iranzo P, Díaz A, Peña A, Estrach MT, Carrera C. Pigmented mammary paget disease mimicking cutaneous malignant melanoma. J Am Acad Dermatol. 2015;72(4):e97–e98. 10.1016/j.jaad.2015.01.015 25773433

[20] D’Aiuto G , Del Vecchio S, Mansi L, D’Aprile M, Botti G, Salvatore M. Malignant melanoma of the nipple: a case studied with radiolabeled monoclonal antibody. Tumori. 1991;77(5):449–51. 10.1177/030089169107700517 1664156

[21] Duman N , Erkin G, Gököz Ö, Kayikçioğlu AU, Celik I. A rare variant of Braf negative melanoma originating from nipple with comparison of the literature data: case report. Turkiye Klinikleri J Dermatol. 2015;25(1):20–3.

[22] Kinoshita S , Yoshimoto K, Kyoda S, Hirano A, Shioya H, Kobayashi S, . Malignant melanoma originating on the female nipple: a case report. Breast Cancer. 2007;14(1):105–8. 10.2325/jbcs.14.105 17245004

[23] Mourmouras V , Ambrosio MR, Onorati M, Rocca BJ, Di Mari N, De Luca F, . Melanoma of the nipple. An additional case. Pathologica. 2011;103(1):25–6. 21837923

[24] D;Antonio A , Addesso M, Quaranta R, Cremone C, Cremone L. Malignant melanoma of nipple: a rare case. Breast J. 2017;23(2):233–4. 10.1111/tbj.12713 27783437

[25] Nagata Y , Yoshioka M, Uramoto H, Tsurudome Y, Yamada S, Hanagiri T, . Malignant melanoma of the nipple: a case report. J Breast Cancer. 2018;21(1):96–101. 10.4048/jbc.2018.21.1.96 PMC5880972 29628990

[26] Mounir M , Bazine A, Rahmoune M, Allilou I, Elmoubakkir B, Ennouhi A, . Primary malignant melanoma of the nipple: a case report. Int J Reprod Contracept Obstet Gynecol. 2021;10(12):4553–4.

[27] Giudice L , Vidali S, Antonini P, Nucci E, Naro FD, Incardona LA, . Primary nipple melanoma in a patient with breast cancer: a diagnosis to consider. Radiol Case Reports. 2024;19(12):6359–64. 10.1016/j.radcr.2024.08.156 PMC11462009 39387024

[28] Hines AS , Campbell EH, Reinhart JP, Crum OM, Proffer SL, Brewer JD, . Primary cutaneous melanoma of the breast skin: incidence, characteristics, and management. JAAD Int. 2024;17:15–6. 10.1016/j.jdin.2024.06.003 PMC11402111 39280988

[29] Lin CH , Lee HS, Yu JC. Melanoma of the nipple mimicking Paget’s disease. Dermatol Online J. 2007;13(2):18. 17498437

[30] Viray H , Bradley WR, Schalper KA, Rimm DL, Gould Rothberg BE. Marginal and joint distributions of S100, HMB-45, and Melan-A across a large series of cutaneous melanomas. Arch Pathol Lab Med. 2013;137(8):1063–73. 10.5858/arpa.2012-0284-OA PMC3963468 23899062

[31] Voiculescu VM , Popescu AI, Costache M. Immunohistochemistry for skin cancers: new insights into diagnosis and treatment of melanoma. Cancers (Basel). 2025;17(11):1769. 10.3390/cancers17111769 PMC12153578 40507250

[32] Cassalia F , Cavallin F, Danese A, Del Fiore P, Di Prata C, Rastrelli M, . Soft tissue sarcoma mimicking melanoma: a systematic review. Cancers (Basel). 2023;15(14):3584. 10.3390/cancers15143584 PMC10377019 37509250

[33] Lens MB , Dawes M, Goodacre T, Bishop JAN. Excision margins in the treatment of primary cutaneous melanoma: a systematic review of randomized controlled trials comparing narrow vs wide excision. Arch Surg. 2002;137(10):1101–5. 10.1001/archsurg.137.10.1101 12361412

[34] Haigh PI , DiFronzo LA, McCready DR. Optimal excision margins for primary cutaneous melanoma: a systematic review and meta-analysis. Can J Surg. 2003;46(6):419–26. PMC3211773 14680348

[35] Morton DL , Thompson JF, Cochran AJ, Mozzillo N, Elashoff R, Essner R, . Sentinel-node biopsy or nodal observation in melanoma. N Engl J Med. 2006;355(13):1307–17. 10.1056/NEJMoa060992 17005948

[36] Long GV , Hauschild A, Santinami M, Atkinson V, Mandalà M, Chiarion-Sileni V, . Adjuvant dabrafenib plus trametinib in stage III BRAF-mutated melanoma. N Engl J Med. 2017;377(19):1813–23. 10.1056/NEJMoa1708539 28891408

[37] Kirkwood JM , Del Vecchio M, Weber J, Hoeller C, Grob JJ, Mohr P, . Adjuvant nivolumab in resected stage IIB/C melanoma: primary results from the randomized, phase 3 CheckMate 76K trial. Nat Med. 2023;29(11):2835–43. 10.1038/s41591-023-02583-2 PMC10667090 37845511

[38] Luke JJ , Rutkowski P, Queirolo P, Del Vecchio M, Mackiewicz J, Chiarion-Sileni V, . Pembrolizumab versus placebo as adjuvant therapy in completely resected stage IIB or IIC melanoma (KEYNOTE-716): a randomised, double-blind, phase 3 trial. Lancet. 2022;399(10336):1718–29. 10.1016/S0140-6736(22)00562-1 35367007

[39] Fleming NH , Zaidi SH, Chagpar AB, Gold H, Darvishian F, Pavlick AC, . Impact of age on management of primary melanoma patients: older patients are less likely to receive postoperative treatment. J Surg Oncol. 2013;108(4):283–8.

[40] Weber J , Mandala M, Del Vecchio M, Gogas HJ, Arance AM, Cowey CL, . Adjuvant nivolumab versus ipilimumab in resected stage III or IV melanoma. N Engl J Med. 2017;377(19):1824–35. 10.1056/NEJMoa1709030 28891423

[41] Eggermont AMM , Blank CU, Mandala M, Long GV, Atkinson V, Dalle S, . Adjuvant pembrolizumab versus placebo in resected stage III melanoma. N Engl J Med. 2018;378(19):1789–801. 10.1056/NEJMoa1802357 29658430

